# Lepromatous Leprosy with Vitiligo, a Clinical Diagnostic Challenge

**DOI:** 10.4269/ajtmh.22-0201

**Published:** 2022-05-09

**Authors:** Harpreet Singh Pawar, Harish Kumar Sagar, Ankur Singh

**Affiliations:** ^1^ ICMR-National JALMA Institute for Leprosy & Other Mycobacterial Diseases, Agra, India;; ^2^ Pathkind Laboratories, Agra, India

Leprosy, a neglected tropical disease caused by *Mycobacterium leprae*, remains a public health problem in India with continued transmission despite its elimination at national level with prevalence of less than 1 per 10,000 population. The clinical diagnosis requires presence of at least one of the three cardinal signs of leprosy: hypopigmented or reddish patch(s) with definite sensory loss, thickened peripheral nerve(s) with impairment of sensations, and the skin smear positive for acid-fast bacilli.^1^ Another condition carrying similar stigma is vitiligo affecting melanocytes in the epidermal basal layer. The presentation of vitiligo is hypochromic to achromic patches and may pose a challenge in clinical diagnosis of leprosy based on its first and most common cardinal sign.

**Figure 1.**
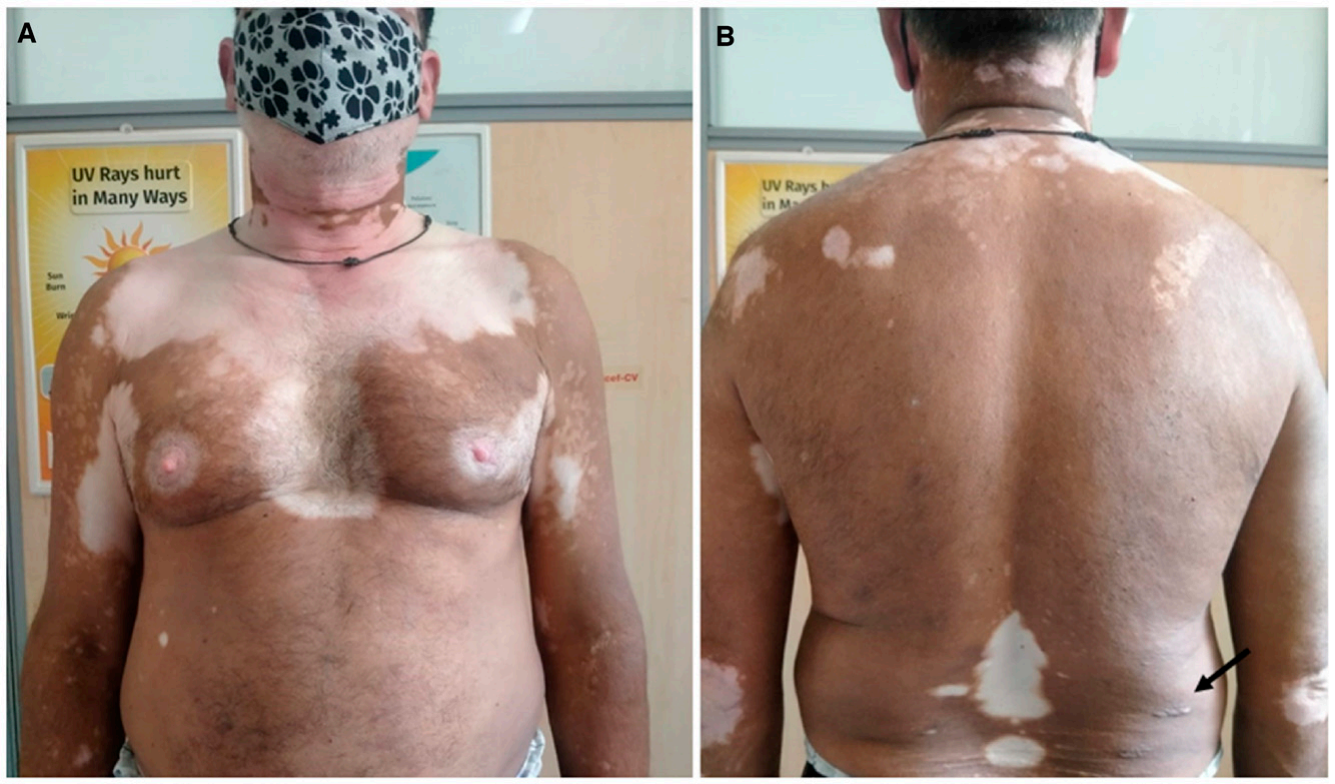
(**A**) Bilateral symmetrical nonsegmental vitiligo. (**B**) Infiltrative lesions of lepromatous leprosy. This figure appears in color at www.ajtmh.org.

**Figure 2.**
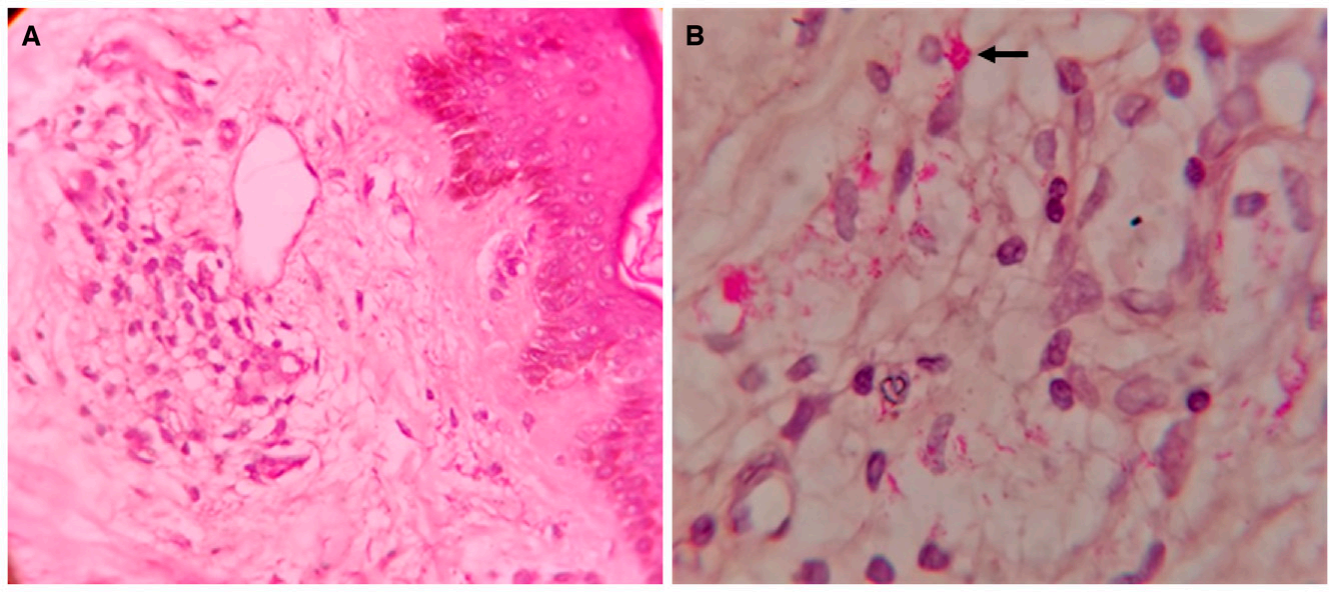
(**A**) Degenerative changes in keratinocytes and increased Langerhans cells with thickening of basement membrane (hematoxylin and eosin stain, 10×). (**B**) Large numbers of acid-fast bacilli deposited in clumps (globi; Fite-Faraco stain, 40×). This figure appears in color at www.ajtmh.org.

A 40-year-old man presented to our outpatient department with complaint of a chronic nonhealing ulcer on the plantar aspect of the right foot. There was no history of hypertension, diabetes, or peripheral vascular disease. The patient was a nonsmoker and had no family or contact history with leprosy patients. The examination revealed large (> 10 cm), generalized, bilaterally symmetrical depigmented macules of 3 years’ duration, diagnosed as nonsegmental vitiligo. Erythematous ill-defined swelling over ear lobes, hypoaesthetic area over the vitiligo patch, infiltration on the back and hypoaesthesia in the gloves and stocking pattern were also noted (Figure 1). The peripheral nerves were bilaterally enlarged but nontender. The Ziehl-Neelsen staining of slit skin smears from three sites and average bacteriological index was 2.3. Figure 2 shows hematoxylin and eosin and Fite-Faraco staining for acid-fast bacilli on punch biopsy from the dorsum. The diagnosis of lepromatous leprosy was made and multibacillary drug therapy was started along with wound management. The patient was referred to a specialty vitiligo clinic.

The present case posed a diagnostic challenge in a patient where the suspicion of leprosy was low and who presented at our center for nonhealing ulcer due to loss of protective sensation. The presence of achromic vitiligo patches obscured the recognition of typical hypoaesthetic patches of leprosy, resulting in delayed cardinal signs–based clinical diagnosis. Previous studies also show increased prevalence of vitiligo in leprosy cases; however, the mechanism of the association is not clear.^2^ Aberration in immune response with autoantibodies is a factor common to both the conditions.^3^^,^^4^ Patients with vitiligo and symptoms of sensory motor impairment should also be screened for Hansen’s disease in endemic areas.
